# Retention of Ti Si snap versus locator attachments with retention sil in two-implant retained mandibular overdentures: an in vitro study

**DOI:** 10.1186/s12903-025-05625-y

**Published:** 2025-02-28

**Authors:** Dina Mourad, Nesrin A. El-mahrouky, Mohamed A. Abd El-Dayem, Yasser M. Shawky

**Affiliations:** 1 Faculty of Oral and Dental Medicine, Misr International University (MIU), km 28 Cairo Ismailia Road, Cairo, Ahmed Orabi District Egypt; 2 Faculty of Dentistry, Al-Azhar University (Girls), Cairo, Egypt

**Keywords:** In vitro study, Overdenture, Attachments, Retention, Ti Si snap attachment, Locator attachment

## Abstract

**Background:**

Primary retention and progressive loss of retention of various attachment systems are critical elements in appropriate attachment selection; nevertheless, research on attachment retention reveals a broad spectrum of retention values for the same attachment system and between various systems. Accordingly, the aim of this study was to compare the retention of two different types of attachments (Ti Si snaps and locators) in two-implant-retained mandibular overdentures.

**Methods:**

A completely edentulous mandibular educational cast was scanned. An STL file including the implant beds and mucosal space was designed. Two implant analogs were incorporated into each model in the canine area bilaterally, and conventional overdentures were fabricated over two types of implant attachments, the Ti Si snap attachments and the locator attachments, with the use of Retention sil in both groups at the fitting surface of the overdenture. Each group contained five 3D-printed edentulous mandibular models. Retention was measured by using a universal testing machine after the models were subjected to cyclic loading. This measurement was carried out at the time of insertion, after 75,000 cycles (simulating 6 months of clinical use) and 150,000 cycles (simulating 12 months of clinical use). The means and standard deviations of the recorded readings were collected, tabulated, and statistically analyzed.

**Results:**

Student’s t test revealed significant differences between the two groups. The Ti Si attachment group presented the highest retention rate at the time of insertion and after 6 months. However, there was no significant difference between the groups after 12 months. Both groups presented statistically significant changes in the mean retention value over time, as demonstrated by two-way ANOVA (time of insertion > six months > twelve months). Tukey’s post hoc test revealed a nonsignificant difference between six months and twelve months.

**Conclusion:**

Within the limitations of this study, the following conclusions can be drawn:

## Background

Complete dentures are still the first choice of treatment in completely edentulous individuals [1, 2]. Completely edentulous patients usually suffer from numerous health conditions, such as pain, loss of retention and stability, inadequate chewing, poor dietary habits and nutritional intake, nausea, poor aesthetics, and speech problems, which influence the quality of life of edentulous patients [3–5]. Furthermore, it can influence the shape and height of the mandibular ridge, resulting in an ill-fitting denture with difficulties in stability and retention [6–8].

Implant-retained prostheses can be utilized in the rehabilitation of edentulous patients, providing superior retention, stability, function, and aesthetics, particularly in the mandible. Furthermore, the use of implants for edentulous patients can help preserve existing bone compared with the use of conventional dentures [9, 10].

A dental prosthesis retained by two implants has the potential to enhance the retention, stability, and masticatory function of the prosthesis, prevent further bone resorption, and improve aesthetics, thereby leading to increased patient satisfaction [11]. Many authors consider the standard technique for treating mandibular edentulism to be the implantation of two conventional implants retaining the mandibular overdenture [12].

The use of attachments has been proven to increase the lifespan of implants by improving overdenture support, stability, and retention [13]. Several attachments are used in the process of attaching implants to overdentures, regardless of whether the implants are splinted or unsplinted [14].

One of the most popular attachments is the locator, which was first introduced in 2001. It is available with various vertical heights, demonstrating an ideal prosthetic solution when the vertical space is limited. The locator attachment is designed to facilitate insertion and removal, features double retention, and possesses self-aligning capabilities, hence enhancing its resilience and tolerance for implant divergence up to 40°. This type of attachment is available in various colors with different retention values. Moreover, its repair and maintenance are easy and fast [15].

Recently, Ti Si snap attachment (Credent medical^®^ GmbH & Co. KG, Senden, Germany), characterized by its titanium‒silicone composition designed to provide a snap effect, was introduced. A corresponding female matrix attachment named Retention sil (R.S., Bredent Medical, Germany), comprising polyvinylsiloxane (PVS), has been developed as a silicon matrix attachment for implant overdentures. This attachment replaces the conventional component within the denture base [16]. The Retention sil attachment offers resilience and high tensile strength, effectively securing the prosthesis through mechanical interlocking and frictional contact. Additionally, it demonstrates good shock-absorbing capabilities, ease of repair, and cost-effectiveness [17]. The attachment is available in three options tailored to the desired detachment forces (200, 400, and 600 g/f) to suit varying clinical requirements. Studies indicate that retention silicon-based overdenture attachment with Ti Si snap abutments, which is based on a bollard-like design, represents a suitable matrix product for resilient retention of implant overdentures owing to its favorable biological, physical, and retention properties [18]. Retention sil 600 reduces visit times and follow-up visits, needs confined space in the fitting surface of the overdenture, makes denture insertion and removal easier, especially for elderly patients with limited manual dexterity. In addition, it is used with immediate loading cases to guarantee minimal stress transfer to implants [19]. It has been reported that in cases of insufficient bone height, implants can be angled to optimize the use of available bone. The use of angled Ti Si snap abutments on obliquely placed implants, therefore, helps to adjust the insertion path [20, 21].

Thermocycling is an in vitro process designed to simulate the temperature fluctuations experienced in the oral environment, which can significantly impact the mechanical properties of dental materials and attachment systems. Attachment systems, which are typically composed of polymers, are expected to undergo changes in retentive capacity when exposed to mechanical stress, temperature variations, and chemical reactions [22, 23]. Such changes often occur within the first year of use [24, 25]. This process involves repeatedly cycling materials between two temperature extremes, generally 5 °C and 55 °C, to replicate the thermal stress caused by activities such as eating, drinking, and breathing. Studies have demonstrated that thermocycling can alter material properties, including retention, flexural strength, and surface integrity, thereby providing valuable insights into the durability and performance of dental attachments [26].

Edentulous patients frequently have issues with their mandibular complete dentures, including function, comfort, and stability. Hence, the degree of retention required for adequate denture stability and, ultimately, successful functioning are likely the most significant factors for dentists and patients when they choose an attachment type. However, the retention of Ti Si snap attachment after thermomechanical cycling remains controversial and needs further investigation. Therefore, the aim of the current study was to evaluate the retention of Ti Si snap versus locator attachments used to retain implant-retained mandibular overdentures with the use of retained sil in both groups at the fitting surface of the overdenture. The null hypothesis tested was that there would be no significant difference in the retention of both Ti Si snap and locator attachments in two-implant-retained mandibular overdentures before and after thermocycling.

## Methods

### Sample size analysis

Power analysis used retention as the primary outcome. The results of Reda KM et al. (2022) are as follows [27]. The resulting effect size (d) was 7.42. With an alpha (α) level of 5% and a power of 80%, the minimum estimated sample size was a total of four samples (2 specimens per group). which was increased to 5 samples per group to obtain more reliable results. Sample size calculation was performed via G*Power Version 3.1.2.2.

### Construction of 3D-printed casts

**Fig. 1:**
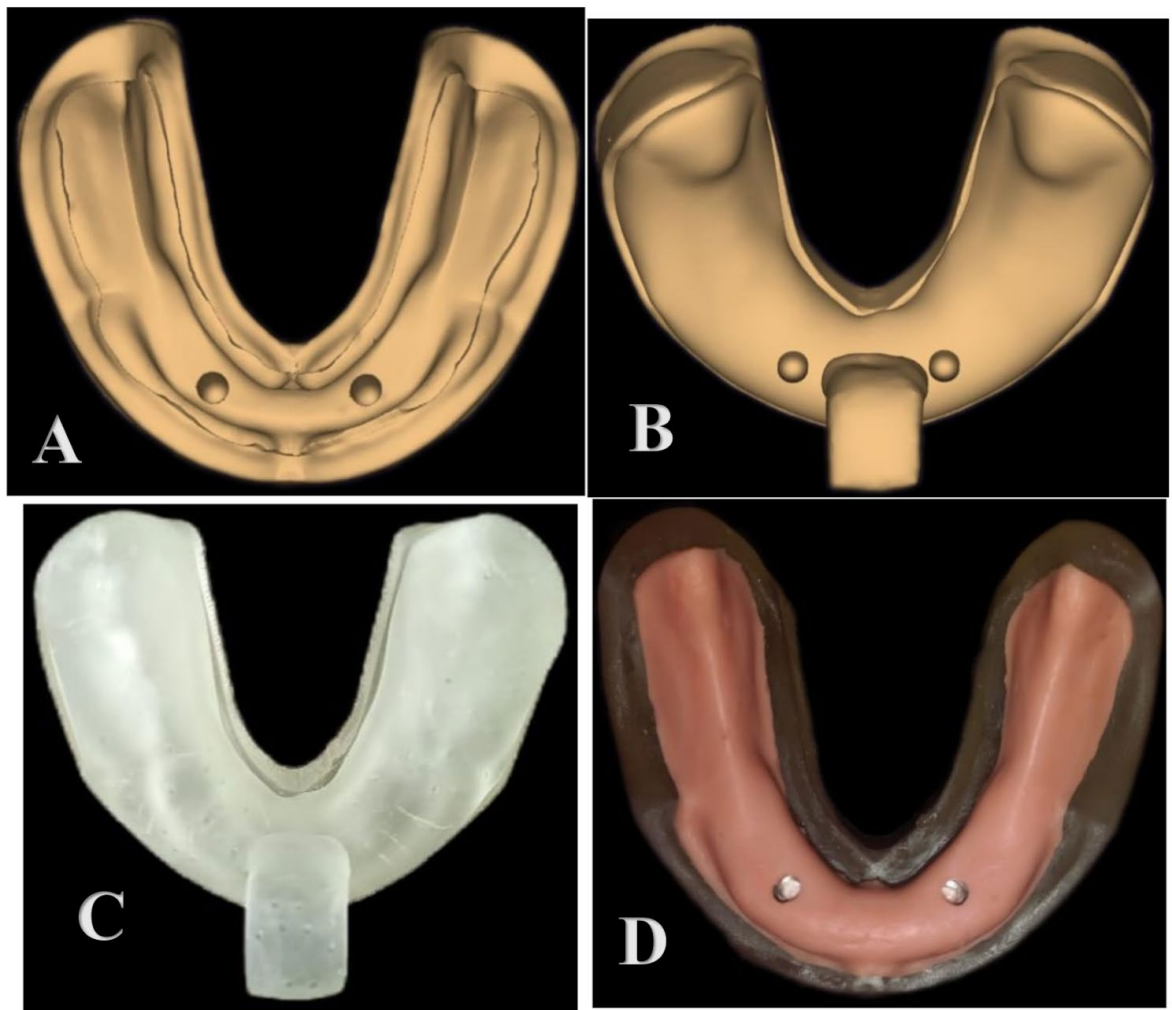
(**A**) 3d-printed mucosa key index; (**B**) STL file with mucosa key index; (**C**) 3d-printed mucosa key; (**D**) mucosa simulation with Multisil-Mask

A scan of both an educational completely edentulous mandibular model and a complete denture made on it was performed via a desktop scanner (Medit IdenticaT500, South Korea). The STL file of the model was created via design software (Exocad Dental CAD, Exocad Inc., Darmstadt, Germany). The STL file of the cast was then imported into Blue Sky Plan software, which is implant planning software. The implant position was determined according to the teeth position of the scanned denture. Implant parallelism was checked via a parallelism tool. A recess for the analog was then created via the implant analog module. These two implant beds presented the intended locations for the two implant analogs with dimensions of 3.5 × 12 mm in the intraformational region (Fig. 1A). A 2-mm thickness was cut from the crest of the scanned model, which was equivalent to the thickness of the mucosa. The STL file was then sent to the 3D printer device (ULTRA 3SP, the Envision TEC (Ferndale, MI) per factory^®^). The cast was printed layer by layer by projecting UV light onto the layers, polymerizing them until the entire cast formed, starting with the base until the crest of the ridge. The raw material used in the fabrication process of the printed model was clear resin (Anycubic, 3D printing UV-sensitive resin, UV wavelength 405 nm; China). A tray for mucosa simulation was then designed over the scanned model, which acts as a key index for mucosa. The special tray was tailored over the model to mimic the viscoelastic behavior of the fibrous mucoperiosteum that covers the residual ridge (Fig. 1B). The tray was then 3D printed from a clear resin (Fig. 1C).

The two analogs (Bredent medical^®^ GmbH & Co. KG, Senden, Germany) were located at their locations in the model and cemented with flowable composite (Dentsply SDR flow, USA).

The mucosa simulation was performed with rubber base material (Multisil-Mask soft, Bredent, Senten, Germany). Multisil-Mask soft is an addition-linking silicone that is directly injected into the mucosa key index from the double-mix cartridge. A 2 mm thick mucosa was produced, which is equivalent to the cut back made during design. (Fig. 1D)

**Fig. 2:**
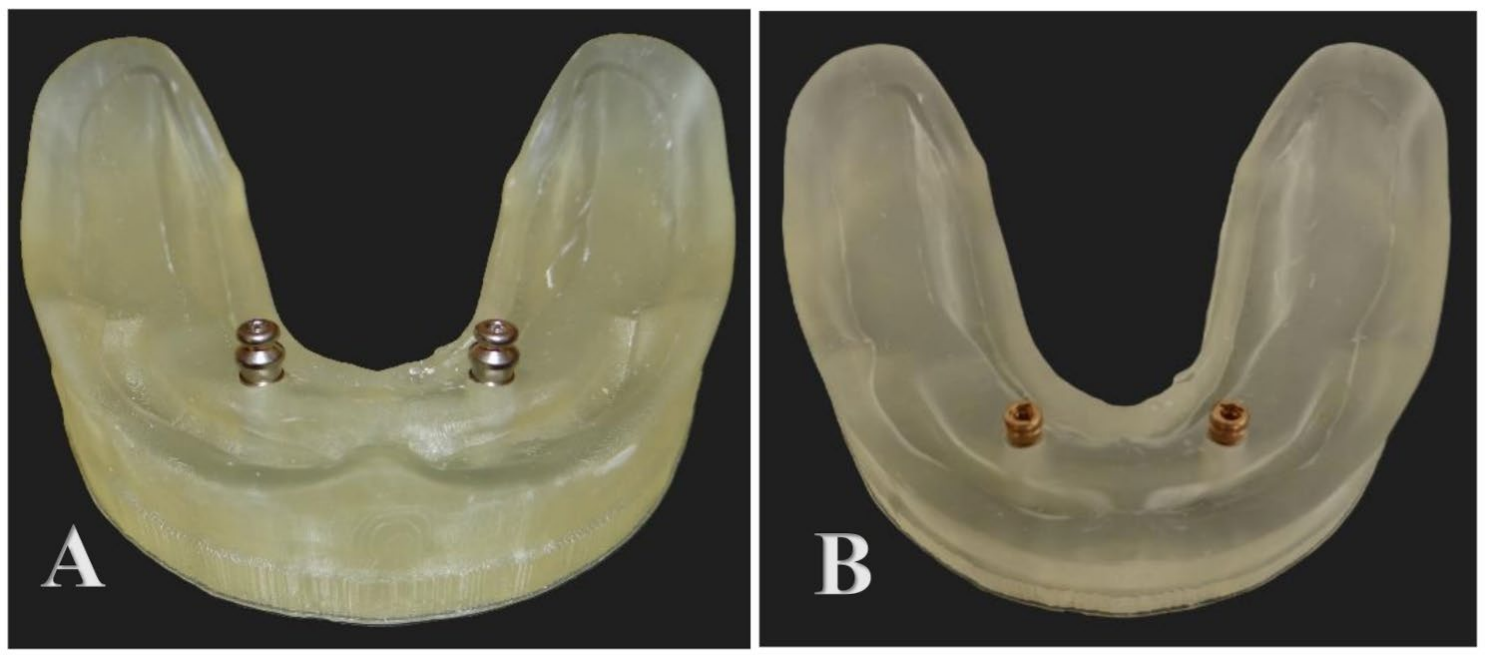
(**A**) Ti Si snap attachment. (**B**) Locator attachment

A total of ten models were 3D printed and then divided equally according to the type of attachment into two equal groups. (Figure 2A and B)

- Ti Si attachment group.
- Locator group.

### Overdenture construction

The model was duplicated using polyvinyl siloxane impression material (Elite HD+, putty soft) and poured into dental stone to create a stone cast for the construction of an overdenture. The overdenture was constructed for both groups and processed in a conventional way utilizing heat-cured acrylic resin. A mold for making duplicate dentures was created using a rubber base impression of the waxed-up trial denture base. The upper portion of the mold was a negative copy of the denture’s polished surface and teeth. The artificial teeth were then put into the corresponding parts of the mold. Ten replica dentures (five dentures in each group) were created by pouring melted base plate wax into the area between the silicone mold and the stone casts. The waxed dentures were flasked with heat-cured acrylic resin (clear heat-cured acrylic resin, Acroston) to create ten replica dentures.

### Application of retention sil

The positions of the attachments in both groups were localized, ground out, and relieved in the fitting surface of the overdenture to create a 1 mm thick layer of silicone material around each attachment in both groups. Escape holes were then made on the overdenture lingually to behave as an exit for the extra material and to avoid extra pressure on the attachment. A sufficient layer of Multisil primer (Multisil-Primer 5 ml, Bredent) was applied to the relieved areas and allowed to dry for 3 min.

**Fig. 3:**
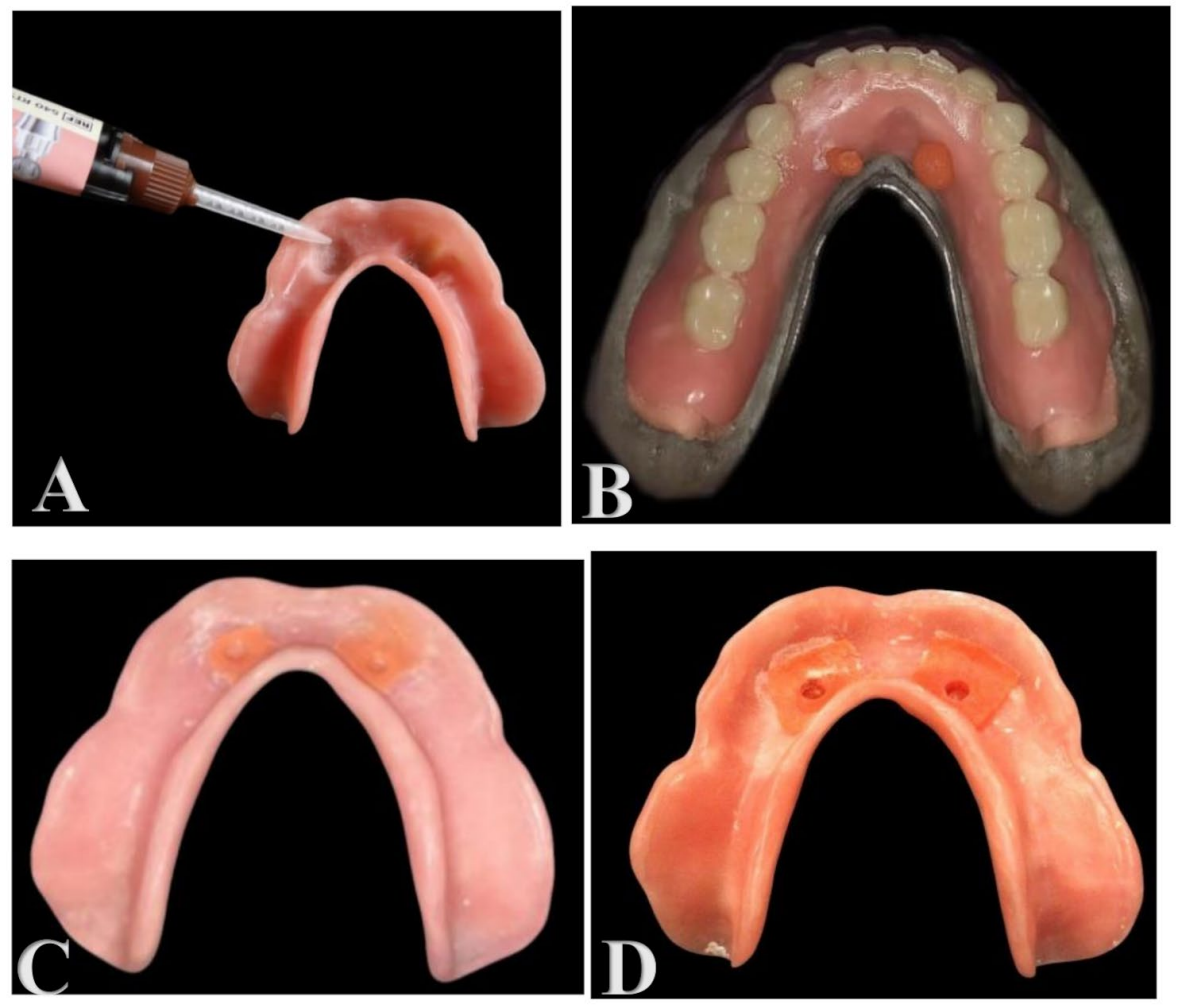
(**A**) Application of the Retention sil 600; (**B**) Escapement of excess material through escape holes; (**C**) Fitting surface of Ti Si overdenture; (**D**) Fitting surface of Locator overdenture

The Retention sil 600 (Bredent Medical GmbH & Co. KG, Germany) was then applied to both the Ti Si attachment group and the Locator group. (Fig. 3A) The overdenture was then installed over the model, with excess material evacuated through escape holes. (Fig. 3B) It was removed and finished with a silicon cutter after setting in both groups. (Figure 3C and D)

### Chewing simulation

The ROBOTA chewing simulator features four chambers that concurrently replicate vertical and horizontal movements under thermodynamic conditions. An upper jackob’s chuck serves as a holding for the vertical screw in each chamber, which can be fixed to the round flat load applicator. (6 cm diameter). Each sample was then placed on the corresponding cast while the upper part of the machine was chucked with the load applicator positioned between the 2nd premolar and 1st molars posteriorly and central incisors anteriorly to facilitate alignment with the loading axis of the machine and proper load distribution. Casts were fixed in a Teflon holder in the lower part of the simulator by cyanoacrylate adhesive. A weight of 5 kg, comparable to 49 N of chewing force, was used. The test was repeated 75,000 and 150,000 times to simulate the 6- and 12-month chewing conditions, respectively.

### Determination of the geometric center

**Fig. 4:**
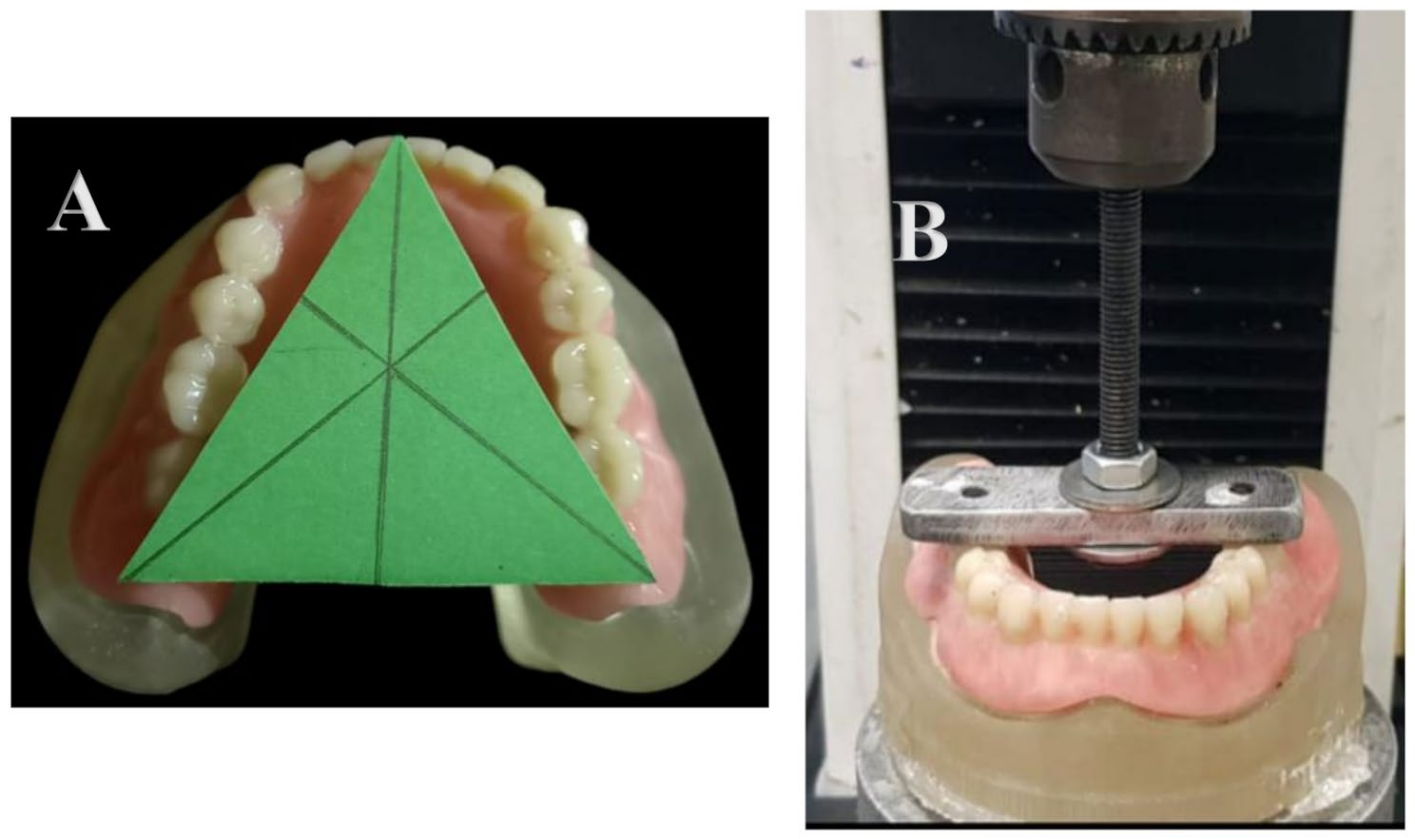
(**A**) Determination of geometric center. (**B**) Bluehill^®^ Lite from Instron Instruments with an inverted T-shaped metal bar glued to the denture (between 5 and 6 bilaterally).

The geometric center of the denture was set at a triangle drawn connected between the retromolar pads and the center of the midline. A cardboard triangle was used, adjusted, and placed to fill the area between the three points (retro-molar pads and midline center). Three lines were then drawn on the cardboard intersecting the triangle’s three angles, which determines the geometric center of the denture [27]. (Fig. 4A)

### Retention measurement

A Bluehill^®^ Lite instrument from Instron Instruments was used to conduct these experiments. Each cast and denture were attached to a lower fixed compartment of a materials testing machine (Model 3345; Instron Instruments Ltd., USA) equipped with a 5 kN load cell, and data were collected via computer software (Bluehill Lite; Instron Instruments). The sample was mounted using a centrally positioned inverted T-shaped metal bar with the horizontal part pointing downward and cemented to the denture (between 5 and 6 bilaterally) to assist in alignment with the machine’s loading axis and proper stress distribution (Fig. 4B). A tensile load with a pull-out mode of force was applied by a wire attached to the upper compartment of a materials testing machine at a crosshead speed of 5 mm/min [29]. The force necessary to completely dislodge the denture was measured in Newtons.

### Statistical methodology

The data are expressed as the means and standard deviations. After the homogeneity of variance and normal distribution of errors were confirmed, one-way analysis of variance was performed, followed by Tukey’s post hoc test if the results were significant. A paired t-test was performed between both groups at each evaluation time. Two-way ANOVA was used to compare the effect of each factor (main group and evaluation time). The sample size (*n* = 5/group) was large enough to detect large effect sizes for main effects and pairwise comparisons, with the satisfactory level of power set at 80% and a 95% confidence level. The results were analyzed via GraphPad InStat (Graph Pad, Inc.) software for Windows. The value of *P* < 0.05 was considered statistically significant.

## Results

**Fig. 5:**
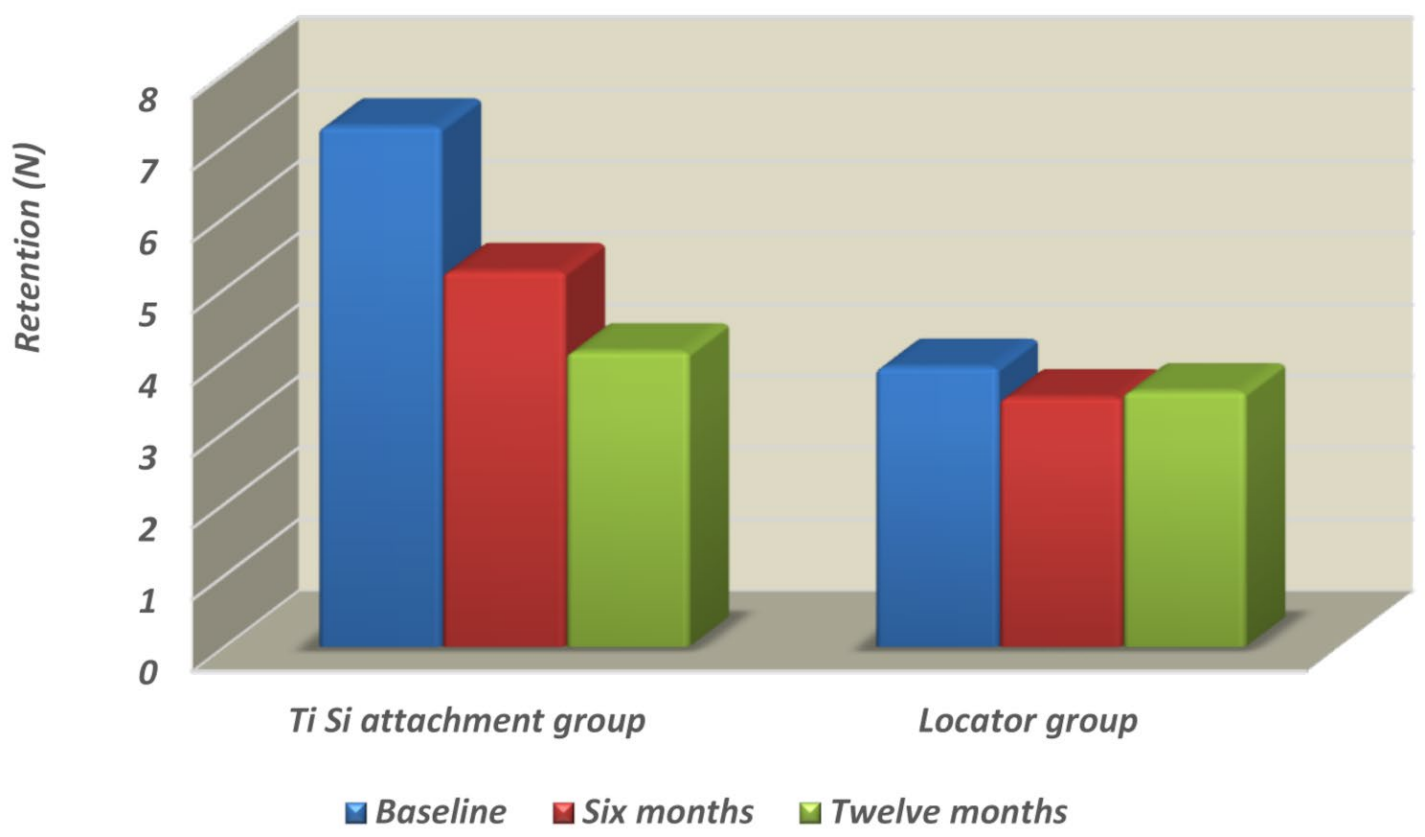
Column chart showing the retention mean values for ***the Ti-Si attachment group*** and ***Locator group*** as a function of evaluation time

**For the Ti Si attachment group**, the highest retention mean value recorded at the time of insertion (7.266 N) was followed by the six-month mean value (5.246 N), whereas the lowest retention mean value recorded after twelve months (4.123 N) was statistically significant, as indicated by one-way ANOVA followed by pairwise Tukey’s post hoc tests (*P* ≤ 0.0001 < 0.05). Table (1) (Fig. 5).

**Table 1:** Comparison of the retention results (mean ± SD) between ***the Ti-Si attachment group*** and ***the Locator group*** as a function of evaluation time

Variable		Ti Si attachment group				Locator group				Statistics
		Mean	± SD	95% CI		Mean	± SD	95% CI		t-test
				Low	High			Low	High	*P* value
***Evaluation time***	***Time of insertion***	***7.266*** ^***A***^	0.269	7.031	7.502	***3.907*** ^***A***^	0.992	3.038	4.777	< 0.0001*
	***Six months***	***5.246*** ^***B***^	0.349	4.94	5.551	***3.481*** ^***A***^	0.823	2.76	4.203	0.0023*
	***Twelve months***	***4.123*** ^***C***^	0.997	3.25	4.997	***3.565*** ^***A***^	0.13	3.451	3.679	*0.2494 ns*
***Statistics***	***P value***	< 0.0001*				*0.6451 ns*				

Different letters in the same column indicate significant differences between evaluation times (*p* < 0.05)

*; significant (*p* < 0.05) ns; nonsignificant (*p* > 0.05)

Low: lowest value at each evaluation time

High: highest value at each evaluation time

**For the Locator group**, the highest retention mean value was recorded at the time of insertion (3.907 N), followed by the twelve-month mean value (3.565 N), whereas the lowest retention mean value was recorded after six months (3.481 N); this difference was not statistically significant, as indicated by one-way ANOVA (*P* = 0.6451 > 0.05). Table (1) (Fig. 5).

### Total effect of material group on retention mean value

Regardless of the evaluation time, the difference between the two groups was statistically significant, as revealed by two-way ANOVA (*p* ≤ 0.0001 < 0.05), where the Ti Si attachment group > the Locator group.

### Effect of evaluation time on the retention mean value

Irrespective of the material groups, both groups presented statistically significant changes in the retention mean value over time, as demonstrated by two-way ANOVA (*P* ≤ 0.0001 < 0.05), where the time of insertion > six months > twelve months. Tukey’s post hoc test revealed a nonsignificant difference between six months and twelve months (*P* > 0.05).

## Discussion

Implant-retained overdentures are considered the treatment of choice for people who are entirely edentulous. As financial constraints, biological and technical factors may make therapy with an implant-supported prosthesis or fixed prosthesis contraindicated [30]. Hence, for completely edentulous patients, implants retained with two-stud attachments are the most straightforward, economical, time-saving, minimal surgical risk, minimally problematic prosthetic alternative, and the least maintenance intensive. Therefore, dentists can anticipate long-term neuromuscular benefits when two implants are used in conjunction with an overdenture, taking into consideration that denture retention is the primary concern for all edentulous patients [31].

3D-printed models were manufactured in this study to allow standardization, dimensional stability, and accurate positioning of the implant in addition to reducing the amount of time and money needed [32]. With the assistance of software and 3D printing technologies, the operator can determine any desired details, which include size, angulation, and placement of implant beds [33]. Multisil-Mask was used to simulate the viscoelastic behavior of mucosa because it has the lowest values of permanent deformation, dimensional changes, and viscoelastic qualities [34].

The implants were placed at the canine area bilaterally in the mandible due to increased bone density and decreased surgical risk in this area. Therefore, overdentures have been shown to have a high implant success rate [35].

The overdenture was duplicated via PVS (Elite HD+, putty soft) to ensure standardization of all the samples [36]. Using addition silicones rather than condensation-curing silicones or irreversible hydrocolloid compounds has several benefits. Since addition silicone is precise and dimensionally stable, it can be saved, and the duplicate process can be finished later. In addition, this approach is easier and saves time. Moreover, the addition silicone can be applied multiple times without losing precision and can capture the required detail even in cases where overdenture abutments are present. In addition, silicone impression materials do not require specialized tools, and duplication can be performed in the clinic [37]. Retention sil has been used in both groups as the female component at the fitting surface of the overdenture to allow standardization [38].

Universal testing machines are believed to be trustworthy and useful devices for evaluating retention forces, especially regarding in vitro research. The occlusal, gingival, mesial, distal, facial, and lingual directions are the usual directions in which mandibular overdentures move within the oral environment. Directional pull testing has been recognized as a useful technique for assessing the stability and retention of overdentures concerning in vitro research, as real unidirectional dislodgement forces are uncommon in the patient’s mouth [39]. The geometric center was determined to allow positioning of the inverted T-shaped metal bar and application of a vertical pulling force to measure denture retention [28, 40].

Gupta et al. [41] concluded that the Locator attachment system has fewer challenges, including retention loss and fewer maintenance appointments, as well as fewer soft tissue and periodontal complications than ball attachment. Furthermore, Cakarer et al. [42] reported that the locator system presented better clinical results than ball and bar attachments did in terms of the rate of prosthodontic complications and maintenance of oral function. However, Kleis et al. [43] reported that locator attachments have a higher rate of maintenance than ball attachments do. The Ti Si snap attachment refers to titanium silicone with a snap effect. With just two implants, the high guiding cone of the Ti Si snap abutments provides secure and dependable denture fixation, providing total control over the denture during integration and removal [44].

In the present in vitro study, the Locator group presented the highest retention at the time of insertion, followed by twelve months, whereas the lowest retention occurred after six months. These results were in accordance with those of Arora et al. in 2017 [45], Rostom et al. in 2021 [46], and Chindarungruangrat et al. in 2022 [47], who reported that retention of Locator attachment seemed to decrease with time and thermocycling. As a result of the surface charge changing during repeated insertion-removal cycles, the hardness and surface roughness increase. This causes fine mechanical friction, which increases the value of the retention force [45].

The Ti Si attachment group presented the highest retention at the time of insertion, followed by six months, whereas the lowest retention occurred after twelve months. These findings are in agreement with those of Yılmaz et al. in 2022 [38], who concluded that loss of retention is directly proportional to thermocycling.

In this study, when comparing the changes in retention between Ti Si snap attachment and locator attachment, it was found that Ti Si snap attachment resulted in great changes in retention from the time of insertion to 75,000 cycles (simulating 6 months of clinical use); however, locator attachment resulted in minimal changes in retention from the time of insertion to 75,000 cycles. After 150,000 cycles (simulating 12 months of clinical use), there was a minimal change in retention for both groups.

Therefore, the null hypothesis was rejected at the time of insertion and after 75,000 cycles (simulating 6 months of clinical use); however, it was accepted after 150,000 cycles (simulating 12 months of clinical use).

Research limitations include the absence of human saliva, and the way the overdenture is burdened during function might affect retention values by increasing attachment friction and wear, making it impossible to evaluate retention pressures regarding in vitro investigations that imitate clinical situations. Furthermore, movements of implant overdentures include complex and numerous directions (occlusal, gingival, mesial, distal, facial, and lingual) when placed in the oral environment. This makes it impossible to simulate occlusal wear in laboratory studies via cyclic dislodging forces, which apply dislodging forces of the same magnitude and direction. A directional pull test is a useful tool for assessing the stability and retention of a prosthesis during in vitro studies, even though real unidirectional dislodging pressures are uncommon in clinical situations. Accordingly, randomized clinical trials are highly recommended to compare the clinical retention of implants retained overdentures with that of both attachments.

## Conclusion

Within the limitations of this in vitro study, the following conclusion can be drawn:

- The retention loss of the Ti Si snap attachment over a period of stimulating 12 months of denture use was great. However, it offers comparable retention compared to locator attachment when Retention sil is used in both groups at the fitting surface of the overdenture.

## Data Availability

The datasets used and/or analyzed during the current study are available from the corresponding author upon reasonable request.

## Abbreviations

Ti Si snap attachment: Titanium‒silicone with a snap effect
Retentionsil: Resilient retentive matrix/female silicone material
3D: Three-dimensional
STL: Standard Tessellation Language
PVS: Polyvinylsiloxane
